# Screening for Acute Childhood Malnutrition during the National Nutrition Week in Mali Increases Treatment Referrals

**DOI:** 10.1371/journal.pone.0014818

**Published:** 2011-06-23

**Authors:** Daniele H. Nyirandutiye, Akory Ag Iknane, Amadou Fofana, Kenneth H. Brown

**Affiliations:** 1 United States Congressional Hunger Center, Washington D.C., United States of America; 2 Helen Keller International, Regional Office for Africa, Dakar, Sénégal; 3 Institut National de la Recherche en Santé Publique, Bamako, Mali; 4 Cellule de Planification et Statistique en Santé, Bamako, Mali; Tulane University, United States of America

## Abstract

**Objective:**

To evaluate a pilot intervention designed to integrate mid-upper arm circumference (MUAC) screening for acute malnutrition into the semi-annual Child Nutrition Week (Semaine d'Intensification des Activités de Nutrition, or “SIAN”) activities carried out in June 2008.

**Design:**

A cross-sectional survey was conducted in Kolokani and Nara, two health districts in the Koulikoro region of Mali, 4–5 months after the SIAN, using a population-proportionate, multi-stage random sample of: 1) health centers, and 2) households in communities linked to each of the selected health centers. Caregivers of 1543 children who were 6–59 months of age at the time of the SIAN, 17 community-based volunteers and 45 health center staff members were interviewed.

**Results:**

A total of 1278 children 6–59 months (83% of those studied) reportedly participated in SIAN. Of the participating children, 1258 received vitamin A (98% of SIAN participants; 82% of all eligible children), 945 received anti-helminth tablets (84% of participants; 71% of eligibles), and 669 were screened for acute malnutrition (52% of participants; 43% of eligibles). 186 of the children screened (27%) were reportedly identified as acutely malnourished. SIAN screening covered a significantly greater proportion of children than were examined in both community-based (22% of children) and health center-based screening activities (5% of children) combined during the 4-5 months after the SIAN (P<0.0001). In general, community volunteers and health personnel positively evaluated their experience adding MUAC screening to SIAN.

**Conclusion:**

Integrating MUAC screening for acute malnutrition in SIAN permits the assessment of a large number of children for acute malnutrition, and should be continued.

## Introduction

Mali is a low-income, West African country with nearly 13 million inhabitants [1], 77% of whom live on less than US$2/day [2]. Following a severe, drought-induced food crisis in 2004–2005, the Malian government developed a National Protocol for the Management of Acute Malnutrition in collaboration with several partner agencies. The program guidelines include recommendations for periodic screening of pre-school children and referral of acutely malnourished children to either the nearest health center or inpatient nutrition service, depending on the severity of malnutrition and presence of clinical complications. To enhance the program's coverage, community volunteers were trained in community-based screening, and local health center staff were instructed to conduct active case-finding among all children presenting to the health center for any reason [3].

The Community Management of Acute Malnutrition (CMAM) program was introduced in the Kolokani and Nara districts of Mali in 2007. The program objectives were to provide the aforementioned community-based activities and to link preventive and curative services. Community volunteers were trained to provide nutritional education sessions, screen children for acute malnutrition, using mid-upper arm circumference (MUAC), and refer and follow-up those with acute malnutrition. The program's early experience indicated that fewer than expected numbers of children were referred for treatment, so new approaches were considered to identify malnourished children. In June 2008, the Ministry of Health decided to conduct mass screening for acute malnutrition in the aforementioned districts during the National Child Nutrition Week, known in French as the “Semaine d'Intensification des Activités de Nutrition” (SIAN). SIAN is a week-long campaign for vitamin A supplementation of children 6–59 months of age and early post-partum women and deworming of children 12–59 months. Since 2003, the campaign has achieved >80% coverage and other programs have begun capitalizing on this success to integrate additional health-related activities, such as immunization and bednet distribution [4].

The objectives of the present study were to evaluate the effects of integrating mass screening for acute malnutrition during the SIAN, and to determine the feasibility of further expansion during future SIAN events.

## Methods

### Study setting and population

The SIAN evaluation was conducted in randomly selected health centers and surrounding communities in the Kolokani and Nara health districts. The study population included caregivers of children 6–59 months, community volunteers and health center staff members. The survey was carried out from October 26 to November 14, 2008, approximately 4–5 months after the June 2008 round of SIAN.

### Sampling

The study was designed as a cross-sectional survey using a multi-stage, population-proportionate, random sample. The first stage of sampling involved the random selection of health centers in both districts from a list of those centers in which the CMAM program was operating, taking into account the size of the population covered by each center, as recommended according to the SMART methodology [5]. The second stage involved random selection of one community in the catchment area of each of the selected health centers, using the same methods. Finally, ∼70 households with a child 6–59 months of age were chosen in each community to participate in the caregiver interviews. For the selection of individual households, each community was divided into four quadrants, and houses were numbered along a randomly selected transect of each quadrant. A random start point was then selected from the numbered houses, and houses to the immediate right of the previously selected house were also included until the desired sample size was achieved. A household was defined as “a group of people who live in the same family compound and eat from the same kitchen”. In total, 20 health centers and 20 communities were randomly selected, 11 in Kolokani and 9 in Nara.

The sample size was calculated based on the assumptions that 15% of children 6–59 months would have been acutely malnourished at the time of the SIAN [6] and 50% of them would have subsequently sought treatment at the health center. We chose a sample size that would permit detection of the hypothesized level of service utilization ±10% with 95% confidence, assuming a design effect of 2 (because of the cluster sampling method) and a non-response rate of 10%. A sample size of 1423 caregivers was estimated to be sufficient, so ∼70 children 6–59 months were needed from each community. In cases where the selected community did not have the required number of children, the study team completed the sample from the community nearest to the one originally selected.

### Data collection, processing and analysis

Separate questionnaires were administered to caregivers of children 6–59 months, community volunteers and health center staff members. In the selected households, all caregivers with children 6–59 months were interviewed. For each caregiver identified, one child 6–59 months was randomly selected for inclusion in the study. The child's age was determined by examining a birth certificate or vaccination card, when available, or by using a local events calendar. The child's MUAC was measured, and the caregiver was asked a series of questions on household socio-economic status, participation in the SIAN, subsequent visits to the health center, and participation in other nutrition-related activities in the community and the health center.

Community volunteers were interviewed if they were involved in nutrition activities in their communities and had received training on the national CMAM protocol. Because at least one volunteer per community was to have received training, the evaluation team planned to interview a total of 20 volunteers, by randomly selecting one volunteer in each community.

In the selected health centers, the evaluation coordinator and one supervisor reviewed the list of children who were identified as being malnourished during SIAN and the CMAM register to determine the proportion of these children who returned to the health center following SIAN screening and the outcome of treatment. The evaluation team also interviewed all members of the health center staff who had participated in the June 2008 SIAN and were trained on the CMAM protocol. The team planned to interview three staff members per health center, for a total of 60 interviews.

Thirteen interviewers and three field supervisors were trained to implement the survey and complete MUAC assessments. All interviewers were standardized against a lead anthropometrist to assess measurement accuracy and intra- and inter-observer measurement variability [7]. The six interviewers whose measurements were most accurate and precise were selected as anthropometrists for the duration of the study.

Two data clerks independently entered data using SPSS version 10 (SPSS Inc., Chicago, IL). Any inconsistencies were cross-checked with the original survey form. The data were analyzed using SAS 9.0 (SAS Institute, Cary, NC). Categorical variables, such as household socio-economic characteristics, participation in SIAN, and reasons for not participating, were examined using frequency distributions. Continuous variables, such as age and MUAC measurements, were evaluated by frequency distributions, and descriptive statistics. Following preliminary descriptive analyses, chi-square tests were used to assess relationships between categorical variables, and the McNemar test was used to compare the proportions of children screened at the different examination sites. Finally, stepwise logistic regression models were developed to determine factors associated with participation in SIAN and with utilization of health services. For factors associated with participation in SIAN, only the 1543 children who were 6–59 months during the June 2008 round of SIAN and therefore eligible to participate in the SIAN were considered. Of the total number of eligible children, 1326 were 12-59 months of age during the SIAN, which is the age range of children who were eligible to receive anti-helminth treatment. Analyses were conducted with and without weighting factors to account for the cluster design of the study and the caregivers' other eligible children, and the results did not differ, so the results are reported only with unadjusted values. Finally, forty-five health center staff members from 20 health centers were interviewed. Each health center covered an average of 13 communities and a population of nearly 11,000 inhabitants.

### Informed consent

The study protocol and questionnaires were approved by the Ministry of Health Nutrition Division, the Koulikoro Regional Health Directorate, the National Public Health Research Institute, and the Cellule de Planification et Statistique-Santé. In the communities, oral consent was obtained from the village chiefs, heads of households and child caregivers before the caregivers were interviewed.

## Results

### Description of study populations

Interviews were conducted with 1741 caregivers, of whom 877 were from Kolokani and 864 from Nara. The caregivers' general characteristics are described in **Table 1**. Almost all primary child caregivers were women (99.4%), most of whom were less than 30 years of age (62.9%), married (97.8%), uneducated (77.7%), and employed in either commerce (58.4%) or agriculture (31.5%). The children's ages and other characteristics are shown in **Table 2**.

**Table 1:** Characteristics of caregivers of children 6–59 months who participated in the evaluation survey (N = 1741) and their households.

Variables	Subcategories	N (%)*
*Age (years)*	<20	203 (11.7)
	20<30	892 (51.2)
	30<40	462 (26.5)
	≥40	184 (10.5)
*Sex*	Female	1731 (99.4)
	Male	10 (0.6)
*Number of births*	<4	748 (42.9)
	4–6	623 (35.8)
	7–10	329 (18.9)
	≥11	41 (2.4)
*Number of living children*	<4	966 (55.5)
	4–6	622 (35.7)
	7–10	152 (8.7)
	≥11	1 (0.1)
*Number of children 6*–*59 months*	1	1175 (67.5)
	2	521 (29.9)
	≥3	45 (2.6)
*Marital status*	Married	1704 (97.8)
	Widow	26 (1.5)
	Single	5 (0.3)
	Separated	6 (0.4)
*Education*	No education	1353 (77.7)
	Some schooling (primary and/or literacy training)	236 (13.6)
	Koranic school	126 (7.2)
	Some secondary school or higher	26 (1.4)
*Occupation*	Commerce	1018 (58.4)
	Agriculture	549 (31.5)
	Fishing	79 (4.5)
	Crafts	46 (2.6)
	None	35 (2.0)
	Teaching	11 (0.6)
	Herding	3 (0.2)
*Primary water source*	Open wells	1151 (66.1)
	Pump water	224 (12.9)
	Faucet water	198 (11.4)
	Closed wells	167 (9.6)
	Stagnant water	1 (0.1)
*Primary form of transport*	Carriage (donkey or horse)	719 (50.2)
	Walking	605 (34.8)
	Motorcycle	189 (10.9)
	Bicycle	50 (2.9)
	Public transport	21 (1.2)
	Private vehicle	2 (0.1)
*Primary source of health information*	Radio	437 (25.1)
	Community volunteers	409 (23.5)
	Health agents	385 (22.1)
	Village chiefs	206 (11.8)
	Family members	144 (8.3)
	No one	81 (4.7)
	Women's groups	39 (2.2)
	Television	23 (1.3)
	Friends	17 (0.9)
*Electricity*	Electricity not present	1714 (98.4)
	Electricity present	27 (1.6)

**Table 2:** Description of the study children 6–59 months.

Variables	Male	Female	Total
N (%)	919 (52.8)	822 (47.2)	1741
*Age (months)*	-	-	-
6–11 months	143 (8.2)	129 (7.4)	272 (15.6)
12–23 months	246 (14.1)	213 (12.2)	459 (26.3)
24–35 months	213 (12.2)	209 (12.0)	422 (24.2)
36–59 months	317 (18.2)	271 (15.6)	588 (33.7)
*N (%) with acute malnutrition according to mid-upper arm circumference (MUAC), N = 1740*			
Moderate (MUAC ≥110 and <120 mm)*	30 (3.3)	51 (6.2)	81 (4.7)
Severe (MUAC <110 mm)*	11 (1.2)	17 (2.1)	28 (1.6)
All (MUAC <120 mm)	41 (4.5)	68 (8.3)	109 (6.3)

Seventeen community volunteers were interviewed for the study. Most of the volunteers (88%) practiced agriculture as their main occupation (**Table 3**). Only 18% were exclusively involved in nutrition-related volunteer activities, while 35% were involved both in nutrition activities and one other volunteer task, and 47% were involved in two or more volunteer activities in addition to nutrition-related ones.

**Table 3:** Description of community volunteers participating in the study (N = 17).

Variables	Subcategories	N (%)
*Sex*		
	Male	15 (88)
	Female	2 (12)
*Education*		
	Some schooling	10 (59)
	Completed primary school	1 (6)
	Incomplete secondary school	4 (23)
	Koranic school	2 (12)
*Primary occupation*		
	Agriculture	15 (88)
	Crafts	1 (6)
	Street vendor	1 (6)
*Other, non-health related, volunteer activities*		
	None	3 (18)
	One activity	8 (47)
	Two or more activities	6 (35)
*Other health-related volunteer activities*		
	Nutrition only	3 (18)
	Nutrition +1 activity	6 (35)
	Nutrition +2 or more activities	8 (47)

### Participation in SIAN

According to their caregivers, 83% of the 1543 eligible children 6–59 months of age and 84% of the 1326 eligible children 12–59 months of age participated in the SIAN in June 2008. Of those in the respective age ranges who participated in SIAN, 98% reportedly received vitamin A, 52% were screened for acute malnutrition, and 85% (of those 12–59 months) received anti-helminthic treatment. Thus, 82% of all eligible children were supplemented with vitamin A, 43% were screened for acute malnutrition, and 71% received an anti-helminthic tablet. Overall participation in SIAN and receipt of specific services did not differ by district. Considering only those children who were eligible to participate in the SIAN (N = 1543), factors associated with participation were: caregiver >20 years of age, child >12 months of age, and prior caregiver participation in nutritional education sessions in the community or residence in communities that conducted routine MUAC screening (see logistic regression results, **Table 4**).

**Table 4:** Factors associated with participation in SIAN.

Variable	OR	95%CI	P-Value
*Caregiver age (years)* *			
<20*	1.00		
20 to <30	1.51	1.02; 2.48	0.039
30 to <40	1.60	1.04; 2.74	0.032
*Child age (months)* †			
6–11	1.00		
12–23	1.32	0.90; 1.95	>0.05
24–35	2.03	1.31; 3.08	0.0014
36–59	10.8	7.91; 14.7	0.0011
*Village-based screening*	0.378	0.249;0.574	<0.0001
*Participation in BCC sessions*			
In the community	0.614	0.411; 0.919	0.017

Reference: *<20 years old.

† 6–11 months; child age group is the age at the time of the SIAN. BCC-Behavior change communication in nutrition.

Because it was decided relatively late in the SIAN training cycle to include MUAC screening procedures in the package of activities, not all communities were adequately prepared to conduct these screening activities. In different communities, the percent of children who received vitamin A during SIAN who were also included in MUAC screening ranged from 15–78%.

For those caregivers whose children were eligible, but did not participate in SIAN (N = 265), the reasons given for non-participation were that they were away from the community (55%), lacked information (21%), arrived late for the session (13%), or were too busy (9%) or ill (0.4%). Notably, the children who did not participate were more likely to be malnourished (defined as MUAC <120 mm or presence of edema) at the time of the follow-up survey than those who did participate (OR = 1.7; 95% CI = 1.2, 2.6; p = 0.007).

### Screening for acute malnutrition and utilization of treatment services

**Figure 1:**
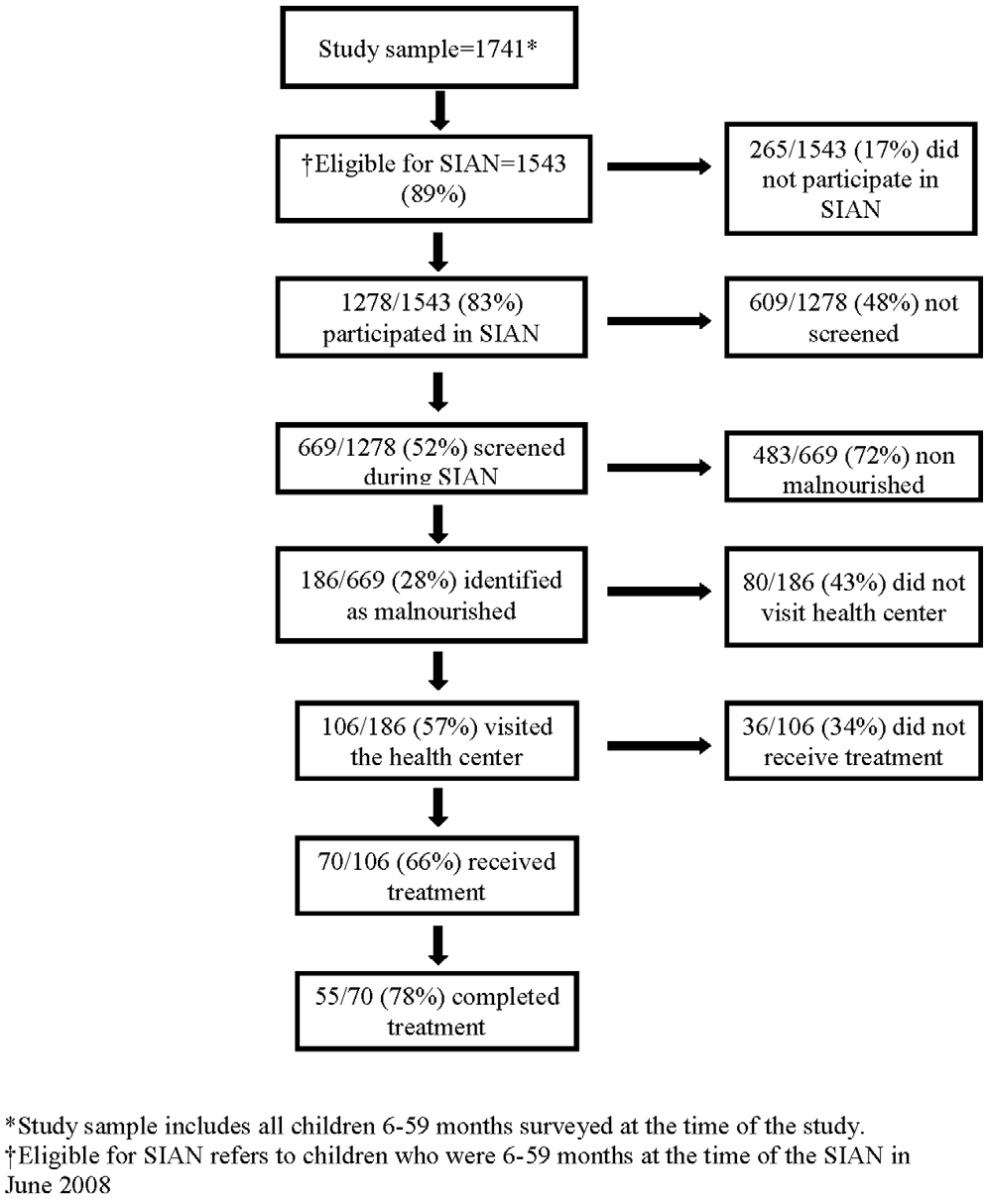
Outcome of screening during SIAN.

As indicated, 669 children were screened for acute malnutrition during SIAN. 186 (27%) of the caregivers whose children were screened stated that their child was identified as being malnourished. 57% of the caregivers whose children were reportedly malnourished said they visited the health center subsequently for further evaluation and treatment. Seventy-eight (74%) of those who attended the health center stated that their children received treatment for malnutrition, and 55 (71%) of those who were treated reportedly completed treatment (**Figure 1**).

**Figure 2:**
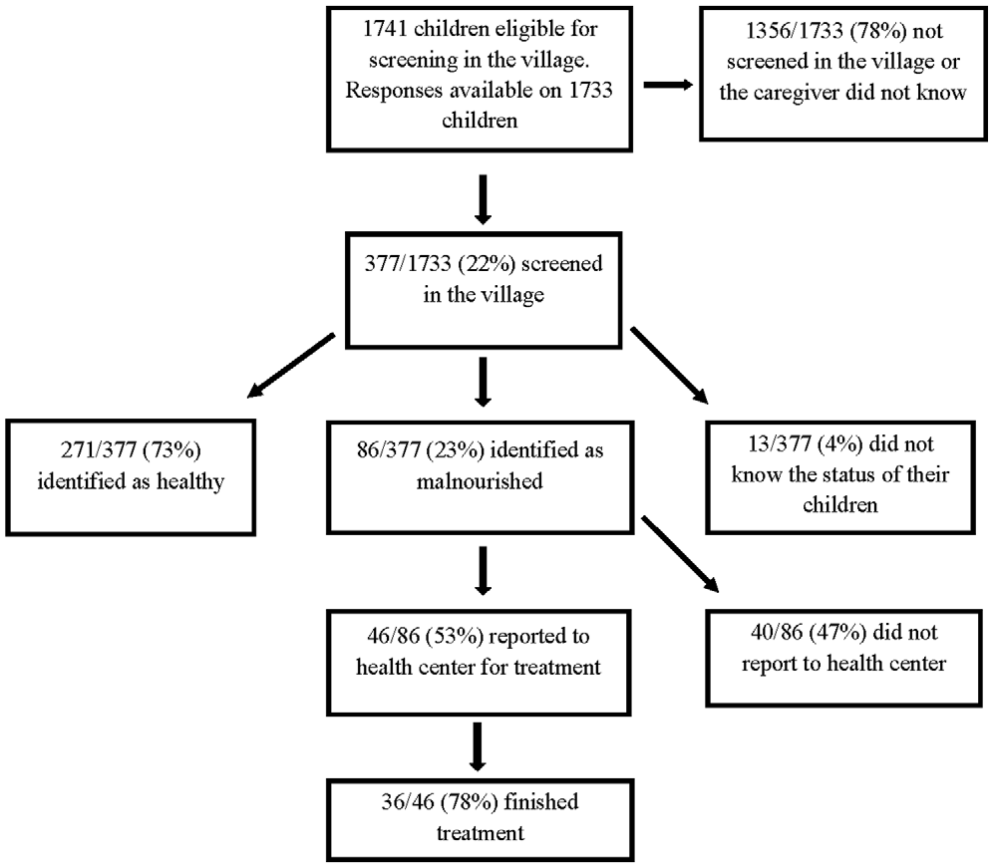
Village screening.

Caregivers were also asked whether their child was screened in the community for acute malnutrition at any time during the 4–5 months since the June 2008 SIAN. Only 377 (22%) responded that their child was screened in the community during that period. Most of the community-based nutritional screening was completed by a community volunteer during routine examinations (80%), and only occasionally during a growth monitoring session (11%) or a community immunization visit by health center staff (9%). Of the 377 children screened in the community, 86 (23%) were reportedly identified as malnourished (**Figure 2**). Fifty-five of these children (54%) were subsequently taken to the health center for treatment, and 42 of the 55 (79%) reportedly finished treatment.

**Figure 3:**
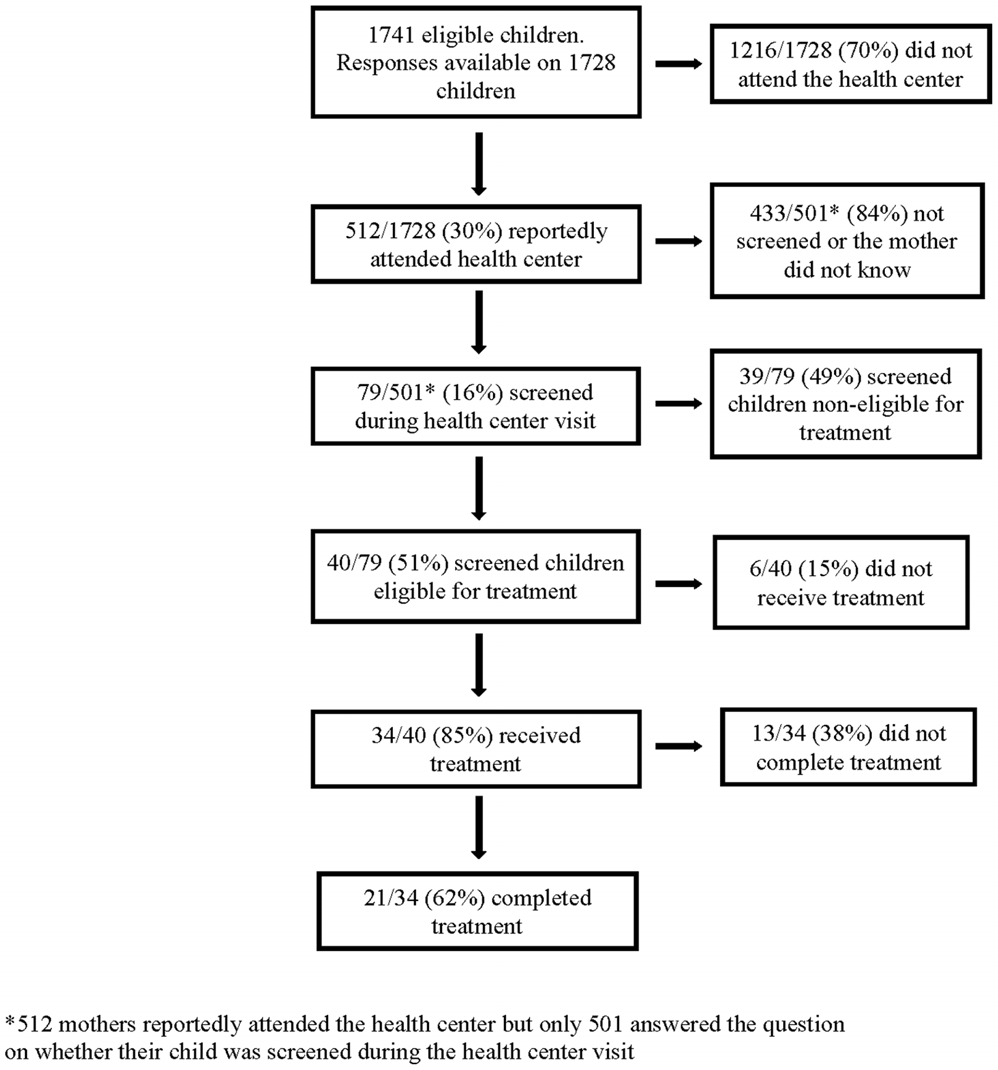
Screening for acute malnutrition at the health center.

30% of caregivers reported that they attended the local health center with the index child during the 4–5 months since the SIAN. Their reasons for attending the health center included child illness (68%), immunization (24%), referral for treatment for acute malnutrition (4%), growth monitoring (0.8%), and maternal pre-natal care (3%). Of the caregivers who attended the health center, only 16% reported that their child was screened for acute malnutrition. **Figure 3** summarizes activities related to screening and management of acute malnutrition at the health center.

Considering both the children's level of participation in the different screening opportunities (SIAN, village and health center contacts) and the frequency with which nutritional status screening actually took place at each of these potential screening opportunities, 43% of all children were screened during SIAN, compared to the 22% of all children who were screened in the community and 5% of all children who were screened at the health center during the 4–5 months following SIAN. SIAN screening covered a significantly greater proportion of children than either of the other two methods during this period (P<0.0001).

We examined the reasons for the relatively low coverage of the routine community-based and health center-based screening. According to caregivers, screening was conducted in all communities, but not all eligible children were included in screening. Community volunteers revealed that they did not always conduct screening because they were either too busy or unaware that they were supposed to perform this task. Health center staff also indicated that not all health centers conducted nutritional screening during community outreach actvities because there were insufficient personnel, the staff members were unaware of this responsibility, or they lacked adequate financial support and/or transportation.

Only the subset of children who visited the health center during the 4–5 months after SIAN had the possibility of being screened at the health center during this period. Factors that favored health center visits were having access to a motorcycle for transportation, having younger children, and prior participation in nutritional education sessions at the health center. Even when the children did visit the health center, their nutritional status was not always assessed. The majority of the health agents (89%) reported that they screened children at the health center just one day a week. The remainder stated that they conducted screening twice weekly (2%), every two weeks (2%), or once a month (7%).

### Treatment for acute malnutrition

The caregivers of nearly all children (∼90%) referred to the health center for acute malnutrition stated that the children received dietary treatment, consisting mostly of Corn Soy Blend, vegetable oil and sugar. The remaining children, most of whom were severely malnourished (MUAC measurement <110 mm), were treated according to the national protocol for severe acute malnutrition. Most children (∼70%) reportedly completed treatment, regardless of where they were originally diagnosed.

### Malnourished children at the time of the survey

1740 children were assessed at the time of the survey using MUAC. The prevalence of acute malnutrition, defined as MUAC <120 mm, was 6.3%. The prevalence of acute malnutrition was significantly higher among girls (8.3%) than boys (4.5%) and among children, 6–11 months (11%) compared to older children (5.4%), as shown in **Table 5**.

**Table 5:** Risk factors for acute malnutrition.

Variables	Odds Ratio	95% CI	P-Value
Sex*			
Male*	1.00		
Female	1.91	(1.32; 2.90)	0.001
Age group (months)**			
6–11 **	1.00		
12–23	1.09	(0.67; 1.70)	0.71
24–35	0.44	(0.24; 0.79)	0.006
36–59	0.09	(0.04; 0.21)	<0.001

Reference categories: *Male, ** 6–11 months

Of the children who were identified as acutely malnourished during the survey, 29 (27%) had ever participated in nutritional screening in the village since the previous SIAN. Forty-six of the malnourished children (43%) had ever attended the health center during the previous ∼4 months; and, of those, only 14 (18%) had been screened for acute malnutrition at the center.

### Health agents' preparation for nutritional screening during SIAN

Fourteen of the 18 health center directors who were questioned about the preparation they received for including nutritional status screening during SIAN felt that they were able to perform the activity, including 13 of the 14 (93%) who had received training at the district hospital. Of those who did not feel that they were able to perform this activity, two said that their centers were understaffed, one did not have adequate transportation, and the fourth declined to answer.

### Acceptability of nutritional screening during SIAN

We also asked caregivers, community volunteers and health center staff to describe their overall impressions of the nutritional screening experience. The vast majority of participating caregivers (98%) found the nutritional screening to be beneficial. Health agents also felt that the screening provided an excellent opportunity to examine more children (87%), supervise the community volunteers (4%), or both (4%). Some also mentioned that screening provided an opportunity to promote CMAM-related services (5%). Fourteen of the 17 volunteers who were interviewed found the activity beneficial because it allowed them to screen many children, but one felt that screening was overly burdensome.

## Discussion

The study results show that inclusion of MUAC measurements to detect acute childhood malnutrition in the portfolio of SIAN activities yielded higher nutritional screening coverage than the routine screening activities carried out in the communities or at the health center during the subsequent 4–5 months. The relatively high rate of screening during the SIAN can be attributed to the fact that the SIAN itself is a successful program with high coverage [4], which led to a correspondingly greater coverage of nutritional screening. Additionally, contact with caregivers during the SIAN provided health workers with an opportunity to inform them about appropriate nutrition practices and available nutritional services, which may have contributed to the subsequent high rate of utilization of health center treatment services for children identified as malnourished. The fact that MUAC screening reached fewer children than other services provided during SIAN may have been due to the fact that the decision to include MUAC screening was reached fairly late in the SIAN cycle, and preparations may have been inadequate. In addition, community mobilization may not have been adequate or conducted in a timely manner to inform caregivers of the screening services. In fact, when caregivers were asked the reason their children were not screened, the majority responded that they were not aware of the additional services. It is also possible that some health centers may not have had enough personnel necessary to carry out the activity. Thus, it is likely that even higher rates of screening coverage could be easily achievable in the future.

There are several advantages of this type of integrated health service delivery. One systematic review revealed that service integration increases uptake when an intervention with low coverage is linked with one that has high coverage [8], which seems to be the case with the current study. Administrative records indicate that vitamin A coverage rates of the Malian SIAN program from 2003 through 2008 [4], [9] were ≥90%. In contrast, several CMAM program reports suggest that screening coverage and treatment of acutely malnourished children are relatively low in the Koulikoro region, where Kolokani and Nara health districts are located. With a total population of 404,299 children 6–59 months of age [9] in the Koulikoro region (∼20% of whom reside in Kolokani and Nara), and a mean 16% prevalence of acute malnutrition, we would have expected to find ∼64,688 acutely malnourished children in this region. However, the most recent quarterly report indicated that only 17,766 children in the Koulikoro region (27.5% of the estimated number of acutely malnourished children) had been treated [10] suggesting that either screening is not taking place regularly or that caregivers of children identified as malnourished are not utilizing the available health services. Indeed, the current results indicate that routine screening coverage was only ∼30% during the preceding ∼4 months, although the majority of caregivers whose children were identified as malnourished used the available services.

The rapid uptake of nutritional screening during the SIAN also may have been due to the coherence of vitamin A supplementation and screening for acute malnutrition, both of which seek to improve the nutritional status of preschool children. This compatibility maximizes efficient use of human resources, thereby reducing costs [8] For example, training on MUAC screening and related data management were incorporated in the health agents' SIAN training; and data collection tools were modified only slightly to incorporate the screening component, thus simplifying the health agents' comprehension. The service integration also may have prompted greater community participation because more of the communities' perceived needs were being addressed. In other settings, integration of vitamin A supplementation into community-directed treatment of parasitic diseases also increased program participation [11].

The SIAN remains the main mode of reaching children 6–59 months with Vitamin A supplementation and deworming services in Mali. Since its inception, the SIAN has been successful reaching more than 90% of the eligible children nationally. Given the current low rate of screening for acute malnutrition in the community and health center, SIAN provides another opportunity to extend nutrition screening and treatment services at minimal additional cost. The advantage of integrating these services includes the ability to use existing human resources and transportation and to combine compatible training, data collection and reporting activities. Fiedler and Chuko [12] showed that the cost of CHDs increased by only ∼5% when an additional service was added. Thus, it is likely that adding nutrition screening during SIAN did not add substantially to the overall cost of the campaign.

Child caregivers who did not take part in the SIAN missed this event because they were transiently absent from their own communities or were either insufficiently informed or poorly motivated to participate. As has been seen in other settings [13], non-participants in the SIAN were more likely to be malnourished at the time of the follow-up survey, suggesting that greater efforts to reach these individuals would be worthwhile.

Possible strategies for increasing SIAN participation include strengthening social mobilization efforts and scheduling post-campaign follow up activities. A previous study in Mali found that vitamin A supplementation coverage during the SIAN was increased by using traditional communication channels [14], so this should be considered in future SIAN social mobilization plans. In the current study, prior participation in nutritional education activities was associated with greater participation in the SIAN, although it is conceivable that caregivers who participated in educational programs were those who were more motivated in general and therefore would have been more likely to utilize nutritional screening services anyway.

The study results show that routine screening in both the communities and health centers remains low, possibly due to understaffing at the health center or insufficient training, motivation and supervision of health agents and volunteers to conduct this activity. Many of the health centers visited during the study did not have the minimum number of staff members (health center directors, nurse aide and matron) specified by the Ministry of Health guidelines. Even where the staffing was complete, many of those who had been trained in the CMAM protocol were not involved in nutritional screening or case management of malnourished children. Moreover, nutritional screening was conducted only sporadically at the health centers; and, according to caregivers, not all children were being screened. This failure to screen all children reporting to the health center represents a missed opportunity for the CMAM program, because it is likely that a disproportionate number of malnourished children are taken to the centers for treatment of associated illnesses. Indeed, nearly half the children who were found to be acutely malnourished at the time of the present survey had attended the health center during the previous four months, but less than one-fourth of them were reportedly assessed for malnutrition at the center.

In the communities, some volunteers did not complete nutritional screening because they reported being too busy. In addition to conducting CMAM activities, 82% of the volunteers were involved in other health and non-health related activities, and all of them had other primary sources of income. Thus, it might be necessary to provide additional incentives to ensure that these individuals are able to dedicate sufficient time to these community service tasks. Also, some volunteers reported that health center staff members had not instructed them to complete nutritional screening. The health center agents also reported that they had insufficient resources to supervise the volunteers adequately.

Approximately half of the children identified as acutely malnourished at the time of the survey had not been previously screened or treated for acute malnutrition in the village or at the health center during the period following the SIAN. This further highlights the need to strengthen community and facility-based nutritional screening. Interestingly, the caregivers reported a considerably higher prevalence of acute malnutrition during the SIAN following nutritional screening than the 6.3% prevalence of acute malnutrition that was actually found at the time of the survey. This may have been due to inaccurate measurements by the community volunteers and health workers during the different screening events, over-diagnosis during the SIAN to exploit available supplementary food supplies, or seasonal differences in nutritional status. Alternatively, the survey anthropometrists may have under-reported acute malnutrition, although this seems unlikely because the survey team had been carefully trained and standardized on MUAC measurements before initiating the field work. With regard to the issue of possible seasonality of acute malnutrition prevalence, the months of June through September, during which children would have been screened for acute malnutrition in the community or health center following the SIAN represent the hungry season, whereas the month of October (during which the survey was conducted) is the beginning of the post-harvest season. Thus, this may explain some of the difference between the reported prevalence during SIAN and that which was measured subsequently.

### Limitations of study

There are several limitations of the present study, which should be recognized. First, the survey was conducted 4–5 months after the SIAN, which may have introduced some recall bias in caregivers' responses. However, vitamin A supplementation has been occurring in Mali since 1998, and caregivers are generally well aware of this activity. At the time of the survey, the caregivers were shown examples of the vitamin A capsules that were distributed during the SIAN to prompt their memory. The reported vitamin A coverage rates are consistent with the results of several national reports^4,9^. MUAC measurements were also completed during the survey, so this should have assisted caregiver recall concerning MUAC screening during the SIAN.

The possibility of recall error may be greater with regard to nutritional status screening at the health center, because health center screening may use either MUAC or weight-for-height assessments, and the caregivers may not have realized that weight-for-height assessments at the health center represented another form of nutritional screening. If this were the case, that might explain some of the apparently low rates of reported screening in the health center. On the other hand, the health staff members themselves also reported that nutritional screening was completed infrequently, so the caregiver reports seem likely to be correct. Finally, the sampling design was limited by the number of clusters selected, which was due to budgetary constraints. However, this limitation would only affect the confidence limits of the results, not the major conclusions of the study.

The integration of nutritional status screening during semi-annual child health weeks (SIAN) provides an opportunity for identifying children with acute malnutrition and referring them for appropriate treatment. The present results suggest that mass screening during the SIAN is feasible and should be continued and expanded to other areas where the CMAM program is being implemented. The study showed that routine screening coverage in the communities and health centers remains inadequate, so these activities should be strengthened, as discussed above. Attempts should be made to identify other individuals who can assist community volunteers in routine screening or to increase the level of financial incentives and/or technical support provided to the volunteers. Other individuals who might be able to perform nutritional screening include school teachers, agricultural extension workers, and leaders of local women's groups. Finally, all children who attend the local health centers for any purpose should be assessed for acute malnutrition. In health centers with inadequate numbers of staff members, efforts should be made to reinforce the staff by recruiting additional personnel to provide the human resources necessary to carry out these activities.
